# Effect of Facebook on the life of Medical University students

**DOI:** 10.1186/1755-7682-6-40

**Published:** 2013-10-17

**Authors:** Hassan Farooqi, Hamza Patel, Hafiz Muhammad Aslam, Iqra Qamar Ansari, Mariya Khan, Noureen Iqbal, Hira Rasheed, Qamar Jabbar, Saqib Raza Khan, Barira Khalid, Anum Nadeem, Raunaq Afroz, Sara Shafiq, Arwa Mustafa, Nazia Asad

**Affiliations:** 1 Sindh Medical College, Dow University of Health Sciences, Karachi, Pakistan; 2 Dow Medical College, Dow University of Health Sciences, Karachi, Pakistan

**Keywords:** Depression, Addiction, Headache

## Abstract

**Background:**

Facebook is a social networking service launched in February 2004, owned and operated by Facebook, Inc. As of June 2012, Facebook reports more than 1 billion active users. Objective of study was to evaluate the effect of Facebook on the social life, health and behavior of medical students.

**Methodology:**

It was a cross sectional, observational and questionnaire based study conducted in Dow University OF Health Sciences during the period of January 2012 to November 2012. We attempted to interview all the participants who could be approached during the period of the study. Participants were MBBS students, while all students of other courses and programs were taken as exclusion criteria. Approximately 1050 questionnaires were distributed to participants. Fifty questionnaires were rejected due to incomplete answers, yielding 1000 usable responses for an approximate 95% response rate. Informed verbal consent was taken from each participant. Study was ethically approved by Institutional Review Board of Dow University of Health Sciences. All the data was entered and analyzed through SPSS 19.

**Result:**

Out of total 1000 participants, males were 400 (40%) and females were 600 (60%). Participants were in the age group of 18–25 years with a mean age of 20.08 years. Most of the participants were using Facebook daily (N = 640, 64%) for around 3–4 hours (N = 401, 40.1%). Majority of them (N = 359, 35.9%) believed that they were equally active on Facebook and in real life while few believed their social life became worse after start using Facebook (N = 372, 37.2%). Most of the participants admitted that they were considered as shy in real world (N = 390, 39.0%) while in the world of Facebook they were considered as fun loving by their friends (N = 603, 60.3%). A large number of participants (N = 715, 75%) complained of mood swings.

**Conclusion:**

Youngsters are willing to compromise their health, social life, studies for the sake of fun and entertainment or whatever satisfaction they get after using Facebook. What we observed in our study was that although majority of our subjects showed multiple signs of Facebook addiction, they don’t realize it and if even they realize it they don’t want to quit Facebook and even if they want to quit, they can’t. Our observance concluded that majority of the users are highly addicted.

## Background

Online social networks are rapidly changing the way human beings interact [1]. Facebook is a social networking service launched in February 2004, owned and operated by Facebook, Inc. As of June 2012, Facebook reports more than 1 billion active users. It is particularly attractive to young adults and over half of member being in the age group of 18-34 [2,3]. University life without Facebook is almost unthinkable and since its inception in 2004, it has quickly become both a basic tool and a mirror for social interaction, personality identity and network building amongst students [4]. Facebook deeply penetrate its users everyday life and now it has become a medium for “change and expression” in every aspect of life [5]. It is one of the incredible inventions of this modern scientific era which fasten every one into its enchantment. It is now available on cellular phones and tablets, anyone can attach to their relative, friend and with news, wherever in the world [2]. There is no lack of examples how the meaning of communication has been changed as a result of social media. Emotion has replace words as a tool for express feelings perhaps most importantly; social media helps to make a society that values frequent communication more than meaning full communication [6].

Beside its huge advantages it has now become a hot topic of debate that either it is a useful invention or an invention with full of hazards. Most of the users don’t realize the negative impact of social media on their life because they are already addicted to it. It has been notified in studies that excessive use of Facebook will make a person to take less interest in his or her surroundings. There is no doubt that excessive use of Facebook affects the real world interaction abilities and communication, while social skills gradually decrease. There is a long list of its negative impact on society as it’s indicated in one study that Facebook is popular mostly among immature people who create bizarre statuses, upload awkward images, and carry out absurd actions, leading to conflict mostly. Facebook addiction is the new term invented by psychiatrists as its addiction will damage the sleeping habits, health and interest in studies and interaction abilities of real life [7].

Pakistani internet users have been on the rise at an accelerated pace, been recognized as one of the emerging nation of internet users. Users of social networking site Facebook in Pakistan have crossed the nine million mark, making Pakistan the 27th most popular country on Facebook. Out of these nine million users, 70% are aged 25 years or younger, while male users are 6.4 million in number and females 2.7 million. Around 44,000 new Pakistani users join Facebook every week [8].

### Objectives

Prior researches provide mixed clues about how Facebook use should influence subjective well-being. Some cross-sectional researches reveal positive associations between Facebook and well-being, other work reveals the opposite. Still other work suggests that the relationship between Facebook use and well-being may be more nuanced and potentially influenced by multiple factors including number of Facebook friends, perceived supportiveness of one's online network, depressive symptomatology, loneliness, and self-esteem. There is no such data available from Pakistan or internationally regarding the effect of Facebook on individual’s health. Whatever is present is lacking in quality and focuses on percentage of its usage, health and psychological effects. Therefore the main reason of our research was to evaluate the effect of Facebook on social interaction, behavior, studies and health of medical students.

## Methods

### Measures

It was a cross sectional study, observational and questionnaire based study conducted during the period of January 2012-November 2012 at Dow University of Health Sciences. Participants were MBBS students. Approximately 1050 questionnaires were distributed to participants. Fifty questionnaires were rejected due to incomplete answers, yielding 1000 usable responses for an approximate 95% response rate. In view of our research objectives, only MBBS students were included, while students of all other courses and programs were excluded. Thus convenience sampling was employed. Sample size was calculated by using open-epi calculator. Informed verbal consent was taken from all participants.

### Questionnaire

Study tool was designed with the help of the Department of Community Medicine, Dow University of Health Sciences. Extensive keyword search was undertaken on Pub med and Google Scholar in order to draft the initial questionnaire. The keywords utilized were “social networking” and “Facebook”. A group of medical students was initially approached and presented with a number of open-ended questions. Output was then incorporated with a thorough review of the literature in order to design the best possible questionnaire. A pretest of this preliminary questionnaire was done on a sample of 15 students in class; questionnaire was revised accordingly in order to ensure best possible form. Final questionnaire demonstrated immediate internal consistency. Conbach’s alpha was calculated for the final data, which came out to be 0.692 for 1st section and 0.648 for 2^nd^ section.

Based on our topic, we made thirty two questions Performa, divided into two sections

Section I

Section I assess the basic demographic characteristics and pattern of using Facebook. It also assessed the psychological and behavioral impact of Facebook.

Q1-Q4 assesses demographic information (name, age, gender and name of college). Q5 and Q6 assess the frequency of internet and Facebook usage. Q7 was about the reason of using Facebook. Q89-Q10 was about effect of using Facebook on social life, time spend with family and friends, and regarding more active either on Facebook or in real life. In Q11 it was asked, do you think Facebook is a source of inspiration and motivation for you. Q12 was about curiosity regarding display pictures. In Q13 it was assess that what are the comments of your friends on your personality in real and on in Facebook. Q14 and Q15 assess the usage if Facebook on late night and waking up at late night especially for logging into Facebook.

Section II

This section evaluates the side effects of Facebook on health and studies.

Q15- Q24 was regarding the side effects of Facebook use (decrease energy level, effect on eye sight and appetite, headache, mood swing, weight problem, headache, irritation and aggressiveness). Q25- Q28 was regarding effect of Facebook on studies. In Q29 it was asked, do you feel loniless inspite of hundreds of Facebook friends while in Q30 it was asked is it difficult for you to spent whole day without using Facebook. Q31 and Q32 were regarding any attempt to reduce Facebook and usage and about future plan of using it. In last Question it was asked whether respondents feel them as an addict of Facebook or not.

### Ethical approval

Research was approved by Institutional review board of Dow University Of health Sciences.

### Analysis

Data from the questionnaire was entered in SPSS (Statistical Package for the Social Sciences) version 19 for analysis and the results were compared. Descriptive statistics formed the basis of the statistical analysis. Frequency and percentages were evaluated for categorical variables. Mean and Standard deviation used for continuous data.

## Result

### Demographics

A total of 1000 questionnaire was correctly filled (response rate 95%). Participants were in the age group of 18–25 years with a mean age of 20.08 years. Majority (N = 600, 60%) consisted of females.

### Usage of Facebook

Majority of participants were using Facebook daily (N = 640, 64%); they were using it for around 1-2 hours (N = 401, 40.1%) and large number of participants used it till late night (N = 411, 41.1%) (Table 1).

**Table 1:** Effect of Facebook on social aspect of medical students of Dow University of Health Sciences

**Serial no**	**Questions**	**Frequency(n = 1000)**	**Percentage (%)**
1	How often do you access internet?	642	64.2
A	Daily	384	38.4
B	2-4 times a week	328	32.8
C	All the time	223	22.3
D	I don’t use it at all	65	0.65
2	How many hours do you use Facebook?		
a	30 min or less	229	22.9
B	1-2 hours	401	40.1
C	2-4 hours	235	23.5
D	More than 4	131	13.1
E	5-6	4	0.4
3	Why do you use Facebook?		
A	It’s a networking site I use it for networking and social purpose	384	38.4
B	To remain in contact with friends and relatives	717	71.7
C	To make new friends	501	50.1
D	For knowledge and educational purpose	189	18.9
E	To spent my spare time when I’m bored	284	28.4
F	Just for fun	362	36.2
4	In your opinion what is the affect of Facebook on you social life?		
A	It’s the same	317	31.7
B	It’s getting better	311	31.1
C	it’s getting worse	372	37.2
5	Is Facebook affected the time that you used to spend with your friends and family?		
A	Yes, it has been decreased	370	37.0
B	Yes it has been increased	15	1.5
C	Decreased a little bit	355	35.5
D	No I still give same time	260	26.0
6	Are you more socially active on Facebook or in real life?		
A	I’m more active on Facebook	319	31.9
B	I’m more active	197	19.7
C	Equally active on both	359	35.9
D	Inactive on either one	125	12.5
6	Do you think Facebook is a source of inspiration and motivation for you?		
A	Yes, it inspires & motivate me	116	11.6
B	No, it demotivates me	160	16.0
C	Sometimes its motivating & sometimes its demotivating	237	23.7
D	Neither motivation nor demotivation	487	48.7

### Reason of using Facebook

Mostly participants used Facebook for keeping in contact with friends and family (N = 717, 71.7%), while (N = 501, 50.1%) people had the reason of making new friends and increase their list of contacts (Table 1).

### Effects of using Facebook

Majority of students admitted that they give much more time to their family and friends before having Facebook in their life as compare to now (N = 370, 37.0%); this has been one of the dilemmas for our society. Most of the people believe that they were socially active both on Facebook and in real life (N = 359, 35.9%) while few considered that their social life became worse after Facebook (N = 372, 37.2%), (Table 1). Majority of participants were clearly rejected the fact that they feel lonely inspite of many Facebook friends (N = 619, 61.9%). Nearly 50% people felt difficult to pass a day without using Facebook while (N = 512, 51.2%) people didn’t feel so (Table 2).

**Table 2:** Effect of Facebook on behavior of medical students of Dow University of Health Sciences

**Serial no**	**Variables**	**Frequency (n = 1000)**	**Percentage (%)**
6	Are you curious about your pictures and spend a lot of time because you want to display a nice display picture on Facebook?		
A	Yes	269	26.9
B	No	474	47.4
C	May be	257	25.7
7	What your friends will comment on your personality?		
A	IN REAL : shy	390	39.0
B	Depressed/Lonely	183	18.3
C	Fun loving	329	32.9
D	Rude	98	9.8
A	ON FACEBOOK : Shy	191	19.1
B	Depressed/Lonely	80	8.0
C	Fun loving	603	60.3
D	Rude	126	12.6
8	How often you use Facebook late night?		
A	Sometimes	411	41.1
B	Always	150	15.0
C	Most of the times	289	28.9
D	Never	150	15.0
9	Do you wake up at night to check your Facebook account?		
A	Yes	229	22.9
B	No	534	53.4
C	I want to	237	23.7
10	Did you feel any difference in your energy level?		
A	Yes I feel more active	95	9.5
B	Yes I feel more lethargic	494	49.4
C	No, I don’t feel any change	411	41.1
11	Did it affect your eyesight?		
A	Yes	513	51.3
B	No	329	32.9
C	Don’t know	158	15.8
12	Do you have headache?		
A	Yes	600	60.0
B	No	400	40.0
13	Do you experience mood swings in daily life?		
A	a) Yes, Sometimes I’m happy and sometimes I’m sad	715	71.5
B	b) No, my mood is always constant	285	28.5
14	Has Facebook affected your appetite?		
A	a) Yes I keep on eating whenever sitting in front of my PC	264	26.4
B	b) Yes I skip my meals because I’m busy on Facebook	238	23.8
C	c) No, fun is fun and food time is for food	498	49.8

Mostly admitted themselves as “shy” in real world (N = 390, 39.0%) but in the world of Facebook they were considered as “fun loving” by their friends 603 (60.3%) (Table 3).

**Table 3:** Effect of Facebook on health and studies of medical students of Dow University of Health Sciences

**Serial no**	**Questions**	**Frequency (n = 1000)**	**Percentage (%)**
15	Do you have GIT problem?		
A	Yes	312	31.2
B	No	688	68.8
16	Is there any change in your weight?		
A	Yes, I gained weight	289	28.9
B	No	361	36.1
C	Yes, I lost weight	350	35.0
17	Does your posture causes backache to you?		
A	Yes	690	69.0
B	No	310	31.0
18	Do you feel depress when you look at other people’s life style?		
A	Yes	230	23.0
B	No	542	54.2
C	Some times	228	22.8
19	Do you get irritated when anyone disturbs you while using Facebook?		
A	a) Yes, I want to use Facebook with full concentration	225	22.5
B	b) Yes, but only when I’m asked to do anything unimportant	526	52.6
C	c) No, I can do anything while using Facebook	249	24.9
20	It has been found out that many Facebook users are aggressive in nature, does this statement apply on you?		
A	Yes	384	38.4
B	N0	616	61.6
21	Does Facebook have negative effect on your studies?		
A	a) Yes	303	30.3
B	b) No. Facebook doesn’t have any effect on my studies	535	53.5
C	c) No, actually it is helpful in my studies	162	16.2
22	Do you use Facebook in your study hours/lectures/workplace		
A	Yes	54	5.4
B	No	550	55.0
C	Sometimes	315	31.5
D	Often	81	8.1
23	Do you use Facebook in your exam & preparation leaves?		
A	Yes	152	15.2
B	No	501	50.1
C	Sometimes	347	34.7
24	Did Facebook affect your GPA?		
A	Yes, it has been decreased	266	26.6
B	Yes, it has been increased	89	8.9
C	No it’s the same	645	64.5
25	Inspite of many Facebook friends do you still feel lonely?		
A	Yes	381	38.1
B	No	619	61.9

Mostly admitted themselves as “shy” in real world (N = 390, 39.0%) but in the world of Facebook they were considered as “fun loving” by their friends 603(60.3%). Mostly participants also complaint of mood swing (N = 715, 71.5%) (Table 2).

### Health effects

Mostly participants complain about headache (N = 600, 60%) and eye sight problem due to excessive usage of computer and mobile devices for operating Facebook. After start using Facebook, many of them also feels change in their work potential which decrease gradually (N = 51.3, 51.3%) (Table 3).

Majority of them didn’t notice any effect on their appetite 498 (49.8%) and on weight 361 (36.1%). More than half of participants suffered from backache due to postural changes 690 (69%) (Table 3).

When question about disturbance was asked, mostly (N = 526, 52.6%), responds that they get irritated when someone asked them to do any thing unimportant during Facebook surfing. A large number of participants disagrees the fact that Facebook addicts were aggressive in nature (N = 616, 61.6%). Nearly half of the respondents thought Facebook didn’t impose any negative impact on their personality (N = 535, 53.5%) (Table 3).

### Effect on studies

Majority of participants denies any effect of Facebook usage on their studies (N = 535,53.5%) and GPA (Grade Point Average) (N = 645, 64.5%) (Table 3).

### Future plans

When asked “What is your future plan regarding Facebook?” most interviewees responded with “I’ll keep using it in future life”. Mostly users didn’t consider themselves as addicted of Facebook (Table 4).

**Table 4:** Represents the past experiences and future plans of medical students regarding use of Facebook

**Serial no**	**Question**	**Frequency (n = 1000)**	**Percentage (%)**
26	Do you feel difficult to spend your day if you can’t log in to your account for an entire day?		
A	Yes	194	19.4
B	No	512	51.2
C	Most of times	294	29.4
27	Do you ever try to reduce your time on Facebook?		
A	Yes, I try but couldn’t succeed	326	32.6
B	No I didn’t try	310	31.0
C	I tried and I succeed	364	36.4
28	What is your future plan regarding Facebook?		
A	I’ll keep using it in future life	722	72.2
B	I’ll stop using it	68	6.8
C	I’ll decrease my time on Facebook	184	18.4
D	I’ll increase my time	26	2.6
29	In your opinion do you think you are a Facebook addict?		
A	Yes	320	32.0
B	No	674	67.4

 Rest of the comparison and frequencies were in Tables.

## Discussion

Within a relatively short time span, Facebook has revolutionized the way people interact. Although a number of studies aimed at assessing the behavioral and psychological impact of Facebook have been published, this is the 1^st^ article from Pakistan that attempt to elucidate the behavioral, heath and psychological effect on Medical students.

Social media as a medium of communication has been continuous to grow around the globe with more than 1 billion users. Each Facebook user has an average of 130 friends who have access to their postings, which may be available to friends of friends or public, depending on the user’s privacy settings. Habitual use of Facebook and its integration into daily life indicated that it has now become an indispensable tool for social, capital and communication with large number of people.

Over past 5 years, social media sites like Facebook have become a central, virtually unavoidable medium for social interaction. Social media sites were particularly attractive to young adults of 18–25 years age group. Findings was also consistent with the data of other studies [9,10]. This age group usually comprises of individuals who were just at the beginning of their educational and professional career and wanted to develop their professional identities.

It was our principle finding that mostly people daily visit their Facebook timeline to update and check or edit their profile, These findings were much higher as compare to previous studies [3,9].

Our study highlighted that 71% of respondents were suffered from mood swing and depression which was much higher in contrast to another studies [11,12]. These preliminary findings did not claim the usage of Facebook as a source of depression; as diagnosis of depression comprised of symptoms and pattern over time with clinical evaluation. Reason of these findings might be excessive use of Facebook by which one encounters relationship changes, luxurious styles and success of other users which put users into a mode of depression. It is the most common health impacting problem of young generation and due to this new invention depression among students has increased up to 56% in the last six years [12].

Mostly users spend 1–2 hours daily on Facebook, same as indicated in a study conducted by Ellison et al. [13]. Most of them used it to keep in touch with friends and relatives [13]. This was demonstrated through the fact that most commonly included information on member’s profile was likely regarding to their professional career or about educational history. (e.g. about their high school).

Statistically our research indicated that there was 50–50 response regarding curiosity, half were curious about uploading attractive pictures while half didn’t [14,15].

Despite the name “Social networking” most users activity on Facebook were self focused, but in our study it was also reported that mostly people didn’t regard Facebook as a source of self motivation or self esteem, only small number of people had claimed that Facebook was a source of inspiration for them which was contrary to past study [16].

Addiction to Facebook is one of the major complain of young generation. When asked about addiction, mostly denies but on contrary when they were asked about disturbance created by social networking sites in their life, mostly claims that Facebook had ruined their social life and now they spent less time with their loved ones. These observations were same as shown in a past study [7]. This might be due to reason that mostly users were so active in editing and constructing themselves on these sites as they entirely forgot their real life problems, necessities and responsibilities [17].

Facebook and other networking sites also give shy people a way to socialize which might otherwise be lacking altogether. It was evaluated in our study that participants who show unwilling-to-communicate or shy in their real life, had considered by their friends as fun loving in Facebook world. These findings were contradicted to past study [18].

Every invention has its both negative and positive effects, same case was with Facebook, and it also has a negative impact on the life of human beings. Mostly its hazardous effects were same as of internet or computer like headache, backache, gain or loose in weight and eye problems [19,20].

### Strength and limitations

The strength of our study lies in interviewing large number of medical students regarding behavioral, psychological and health effects of Facebook. Previous studies have targeted different communities or age groups; we use a medical university for extracting the data in order to provide a different perspective with regard to our study topic. All attempts were made to ensure that the data collected was reliable and the methods were reproducible. However, our study was also not free from limitations. The most important limitation was that it just occurs in one medical university which comprises of three medical colleges. Although, these medical colleges consist of a heterogeneous population coming from different backgrounds, they cannot be used to predict the overall situation in the country. Furthermore, convenient sampling was employed, which may have led to selection bias, and hence is not truly representative of the population under study. However, since this was just an observational study, the sampling method did seem to fulfill its purpose.

### Future researches

Although these findings raise numerous future research questions, few stand out as most pressing. Do these findings generalize? We concentrated on students of only one medical university.

•This implies that further studies should be conducted on a larger scale, with a more diverse set of institutes in order to minimize bias and for better generalization.

•Future research should also examine whether these findings generalize to other online social networks.

## Conclusion

The human need for social connection is well established, as are the benefits that people derive from such connections. On the surface, Facebook provides an invaluable resource for fulfilling such needs by allowing people to instantly connect. It also has been evaluated that online social networking may interfere with physical activity, which has cognitive and emotional replenishing effects and trigger damaging social comparisons. Any social network has two sided picture, on one hand where it a way of communication between friends and families on the other there are not only bad effects on youth but also great loss of precious time. So it should be used for creative and productive work not as a harmful tool for health and real life relations.

## Competing interest

The authors declare that they have no competing interest.

## Authors’ contributions

HF and HP had substantially contributed in conception, design and acquisition of data. HMA did analysis and interpretation of data and manuscript drafting. SS, IQ, MK, NI, HR, QJ, SR, BK, AN, RA, SS, AM and NA did data collection and critically review the manuscript. All authors read and approved the final manuscript.

## Authors’ information

Hassan farooqi = Final year student of Dow University of Health sciences

irockzzz@hotmail.com

Hamza patel = Final year student of Dow University of Health sciences

hamzapatel_90@yahoo.com

Hafiz Muhammad Aslam = Final year student of Dow University of Health sciences

coolaslam8@hotmail.com

Shafaq saleem = Final year student of Dow University of Health sciences

drshafaqsaleem@gmail.com

Iqra ansari = Final year student of Dow University of Health sciences

ansari_iqra@yahoo.com

Mariya khan = Final year student of Dow University of Health sciences

mariya.libra@gmail.com

Noureen iqbal = Final year student of Dow University of Health sciences

noureen_4best@yahoo.com

Hira rasheed = Final year student of Dow University of Health sciences

hira_445@hotmail.com

Qamar jabbar = Final year student of Dow University of Health sciences

qamar.jbar@hotmail.com

Saqib raza = Final year student of Dow University of Health sciences

saqib.raza31@yahoo.com

Barira khalid = Final year student of Dow University of Health sciences

speak_gently@hotmail.com

Anum nadeem = Final year student of Dow University of Health sciences

ana17_nadeel@hotmail.com

Raunaq afroz = Final year student of Dow University of Health sciences

rounaqafroz@yahoo.com

Sara shafiq = Final year student of Dow University of Health sciences

savinrao@hotmail.com

Arwa mustafa = Final year student of Dow University of Health sciences

aquagurl_15@hotmail.com

Nazia asad: Final year student of Dow University of Health sciences

nazya.scorpion@gmail.com
